# Identifiability of Large Phylogenetic Mixtures for Many Phylogenetic Model Structures

**DOI:** 10.1007/s11538-026-01782-5

**Published:** 2026-10-23

**Authors:** Bryson Kagy, Seth Sullivant

**Affiliations:** 1 Texas State University, San Marcos, United States; 2 North Carolina State University, Raleigh, United States

**Keywords:** Phylogenetics, Identifiability, Jukes-Cantor, Equivariant models, Kruskal’s theorem, Mixture models

## Abstract

Identifiability of phylogenetic models is a necessary condition to ensure that the model parameters can be uniquely determined from data. Mixture models are phylogenetic models where the probability distributions in the model are convex combinations of distributions in simpler phylogenetic models. Mixture models are used to model heterogeneity in the substitution process in DNA sequences. While many basic phylogenetic models are known to be identifiable, mixture models in generality have only been shown to be identifiable in certain cases. We expand the main theorem of [Rhodes, Sullivant 2012] to prove identifiability of mixture models in equivariant phylogenetic models, specifically the Jukes-Cantor, Kimura 2-parameter model, Kimura 3-parameter model and the Strand Symmetric model.

## Introduction

Phylogenetic models represent the evolutionary relationship among a collection of taxa (short for taxonomic unit, which might be genes, species, or some other level of biological classification). Basic phylogenetic models are constructed by starting with a rooted tree where the taxa are vertices. Taxa closer to the root correspond to more ancestral units in the evolutionary history. Associated to each edge in the tree is a transition matrix. Each transition matrix encodes the substitution probabilities of the resulting characters along that edge. Specifying a substitution model amounts to placing restrictions on the structure of the transition matrices.

In the General Markov model no assumptions are made on the entries of the transition matrices (other than the stochastic assumption), whereas in the Jukes-Cantor model all off diagonal entries are equal so the transition matrices are highly constrained. Models like the Kimura 2-parameter, Kimura 3-paramater, and the Strand Symmetric model sit in between these two extremes.

When using any statistical model in practice, it is desirable if a distribution arising from the model uniquely determine the parameters that produced in, i.e. the model should be *identifiable*. For basic phylogenetic models, these parameters are the tree parameter and the transition matrices. The tree parameter is a discrete parameter, in that there are only finitely many different trees on *n* leaves. On the other hand, the space of possible transition matrices forms a continuous parameter space. Questions of identifiability concern both the uniqueness of the discrete parameters and the continuous parameters as determined from probability distributions produced by the model. In this paper, by identifiable we mean *generically identifiable*, that is, the parameters that produce a distribution are identifiable except possibly for a low dimensional subset of the parameter space. All the basic phylogenetic models are known to be generically identifiable by classic results in the literature (Chang 1996).

This paper concerns the identifiability of mixture models, which are more complex models which use the basic phylogenetic models on trees as building blocks. In a mixture model, we do not assume that all characters evolve according to the same underlying phylogenetic tree with a fixed set of parameters. Rather there are (hidden) classes, and depending on which class a character belongs to, it evolves under the (basic) phylogenetic model according to that class. Classes might correspond to sites in DNA sequences that evolve at different rates (fast or slow). Across larger regions of DNA, classes could correspond to different genes which evolve according to different trees altogether. Mixture models have been used in situations where the precise nature of heterogeneity across sites in the data is unclear (Huelsenbeck and Suchard 2007). Also, general mixture models often contain phylogenetic network models as submodels. For example, the displayed tree model on a network (also called the network Markov model), is a submodel of a related mixture model, which can be useful for proving identifiability results about phylogenetic network models, as in Ak Englander et al. (2025).

As mixture models have many more parameters than the basic phylogenetic models, questions of identifiability become increasingly more difficult to answer and their analysis requires more advanced tools. Limited work has been done on the identifiability in group-based mixture models for specific numbers of classes and for completely general tree structures. For example, identifiability is known for 2 class mixtures for the Jukes-Cantor and Kimura 2-parameter models (Allman et al. 2011) for the Cavender-Farris-Neyman model and the Kimura 3-parameter model (Hollering and Sullivant 2021). For 3 class mixtures identifiability is only known for the Jukes-Cantor model (Long and Sullivant 2015). Work has also been done on the identifiability of other types of mixture models such as Profile Mixture Models (Yourdkhani et al. 2021), which represent a useful submodel of general mixture models.

Probably the strongest result on identifiability of mixture models, the main theorem in Rhodes and Sullivant (2012) gives conditions under which mixture models under the General Markov model are identifiable. While this result does not allow for arbitrary mixtures with completely unrelated sets of trees, it does cover a large class of sets of trees, including the same tree mixtures which are the mostly commonly occurring. In most settings, these results allow for identifiability in a number of mixture classes well above any that could be used in practice.

All the results mentioned so far consider generic identifiability, which, while a necessary consideration to use for mixture models, does not typically produce identifiability results for submodels. Hence, the main results of Rhodes and Sullivant (2012) only apply to the General Markov model, and not to any of the submodels: Jukes-Cantor (JC), Kimura 2 parameter model (K2P), Kimura 3 parameter model (K3P) and the Strand Symmetric model (SSM). These submodels are called *equivariant* because they have an underlying model symmetry to the assumptions they make on the transition matrices. The goal of this paper is to broaden the tools developed from the main theorem in Rhodes and Sullivant (2012) to also deduce the identifiability of those important submodels.

The outline of this paper is as follows. Before continuing onto the rest of the paper, in the next section we give some important definitions necessary to state the main results of this paper. Theorem 2.6 gives a general framework for proving identifiability results for phylogenetic mixture models and Corollary 2.7 establishes that the JC, K2P, K3P, and SSM all satisfy those conditions. In Sect. 3, background on phylogenetic trees and the Markov, group-based, and equivariant models is given. In Sect. 4, the discrete Fourier transform is introduced, which will be useful to determine the rank of certain matrices in our computations. In Sect. 5, Kruskal’s theorem is introduced which gives bounds on the rank of tensors and will be crucial in the proof of the main theorem. The technical heart of the paper is Sects. 6, 7, and 8. Specifically, in Sect. 6, we prove our general identifiability result showing that if a Markov model μ satisfies four key properties, then the associated mixture models will be generically identifiable. Sections 7 and 8 then prove that the JC, K2P, K3P, and SSM models all satisfy these technical conditions to derive our main result.

## Key Definitions and Statement of Main Results

The goal of this section is to give the necessary definitions so that we can state our main results. This section just covers definitions to help make sense of the results. Further definitions will appear in subsequent sections.

Let *T* be a single phylogenetic tree with *n* leaves. Let μ be a *Markov model*, (for example, JC, K2P, K3P, SSM, or GMM standing for Jukes-Cantor, Kimura 2-parameter, Kimura 3-parameter, Strand Symmetric model, or General Markov Model, respectively). Let $S^{\mu }_{T}$ be the parameter space of continuous parameters for the underlying model μ on tree *T* (which consists of transition matrices for each edge, and a root distribution). Let κ be the number of states of the random variables being considered (κ=4 is most common in this paper). Once we specify these parts of a model, there exists a parametrization $\psi ^\mu _{T}$ which gives the joint distribution of states on the leaves of *T* as a function of continuous parameters which specify the root distribution of the tree and all of the transition matrices on its edges. That is,

$$ \psi ^{\mu }_{T}: S^{\mu }_{T} \rightarrow \Delta ^{\kappa ^n-1} $$

where $\Delta ^{\kappa ^n-1} \subset [0,1]^{\kappa ^n-1}$ is the probability simplex of non-negative real vectors summing to 1. Then the image of $\psi ^{\mu }_{T}$ is the phylogenetic model $M^{\mu }_{T}$.

Given a multiset of *r* phylogenetic trees $T_1, \ldots , T_r$, a phylogenetic mixture model can be defined as the set of convex combinations of distributions from $M^{\mu }_{T_1}$, …, $M^{\mu }_{T_r}$.

### Definition 2.1

Let $\textbf{T}= (T_1, \dots T_r)$ be a *r*-tuple of trees each with *n* leaves. Let $S^{\mu }_{\textbf{T}}= S^{\mu }_{T_1} \times \dots S^{\mu }_{T_r} \times \Delta ^{r-1}$, each with underlying model μ. Then, the parametrization map of the *mixture model* is defined by

$$ \psi ^{\mu }_{\textbf{T}}: S^{\mu }_{\textbf{T}} \rightarrow \Delta ^{\kappa ^n-1} $$

where

$$ \psi ^{\mu }_{\textbf{T}}(s_1, \dots , s_r, \pi ) = \pi _1\psi ^{\mu }_{T_1}(s_1) + \dots + \pi _r\psi ^{\mu }_{T_r}(s_r) $$

with $s_i \in S_{T_i}$, and $\pi \in \Delta ^{r-1}$ is the vector of mixing parameters.

Since there is no order on the combination of trees in mixture model, identifiability on mixture model will have to be defined up to reordering of the trees.

### Definition 2.2

The tree parameters of an *r*-tree mixture model on $\textbf{T} =(T_1, \dots , T_r)$ are *generically identifiable* if for generic choices of $s_1, \dots , s_r$ and π with $s_i \in S^\mu _{T_i}$, and $\pi \in \Delta ^{r-1}$,

$$ \psi ^{\mu }_{\textbf{T}}(s_1, \dots , s_r, \pi ) = \psi ^{\mu }_{\textbf{T}'}(s'_1, \dots , s'_r, \pi ') $$

implies that there is a permutation $\sigma \in \mathfrak {S}_r$ (the symmetric group on *r* letters) such that $\sigma \cdot \textbf{T} = \textbf{T}'$.

### Definition 2.3

The continuous parameters of an *r*-tree mixture model on $\textbf{T} =(T_1, \dots , T_r)$ are *generically identifiable* if for generic choices of $s_1, \dots , s_r$ and π with $s_i \in S^{\mu }_{T_i}$, and $\pi \in \Delta ^{r-1}$,

$$ \psi ^{\mu }_{\textbf{T}}(s_1, \dots , s_r, \pi ) = \psi ^{\mu }_{\textbf{T}}(s'_1, \dots , s'_r, \pi ') $$

implies that there is a permutation $\sigma \in \mathfrak {S}_r$ such that $\sigma \cdot \textbf{T} = \textbf{T},$ and $\psi _{T_i}(s'_i) = \psi _{T_i}(s_{\sigma (i)})$, and $\pi '_i = \pi _{\sigma (i)}$ for i∈[r].

In the identifiability of the continuous parameters of a phylogenetic mixture model, we allow for label swapping of two types. First, there can be label swapping that permutes parameters between two classes of the mixture model that have the same underlying tree. Second, within the set of distribution of a single tree, there might be unidentifiability due to label swapping of the states of hidden variables. That is, the continuous parameters of the mixture model are identifiable provided the only non-identifiability is due to label swapping of the mixture classes, plus the nonidentifiability of the individual tree models.

We must define the class of mixtures that we prove identifiability in. This amounts to restricting to a subset of *r*-tuples of trees.

### Definition 2.4

Let $\mathcal {T}(r,n,k)$ be the set of *r*-tuples of *n*-leaf binary trees $\textbf{T} = (T_1, \dots , T_r)$ such that there exists a tripartition of the leaves *A*|*B*|*C* and for each i∈[r] a vertex $v_i$ in tree $T_i$ such that the induced triparition of the leaves in $T_i$ induced by $v_i$ is *A*|*B*|*C*. Furthermore, we assume that $\#A \ge \#B \ge \#C$ and $\#B \ge k$.

### Example 2.5

Let $\textbf{T} = (T_1, T_2)$ where $T_1$ is the tree on 6 leaves with nontrivial splits $\{123|456, 12|3456, 1234|56 \}$ and $T_2$ has nontrivial splits $\{123|456, 13|2456, 1234|56 \}$. Then $\textbf{T} \in \mathcal {T}(2,6,2)$ since both trees have a common tripartition 123|56|4, and $\# \{5,6\} = 2$.

We now state our main result, which depends on some technical definitions which will appear in later sections. However, this gives us the flavor of the results that can be achieved with these methods, and the fact that certain specific properties need to be proved for a model μ to deduce identifiability for a mixture model. These properties are *standard*, *the rank property*, *the extended rank property*, and *the No Shuffling Property*.

### Theorem 2.6

Suppose that a phylogenetic model μ on κ>2 states satisfies the following properties:

1. μ is standard,
2. μ has the rank property *RP*(*r*, *k*)
3. μ has the extended rank property *ERP*(*r*, *k*)
4. μ has the No Shuffling Property
5. $$r \le \kappa ^{k -1}$$, and
6. n≥2k+1.

Then both tree parameters and the numerical parameters of $M^{\mu }_{\textbf{T} }$ are generically identifiable in the class of trees $\mathcal {T}(r,n,k)$.

### Corollary 2.7

Let μ be any equivariant model on κ=4 states. Suppose that n≥2k+1 and $r \le 2^k - k$. Then both tree parameters and the numerical parameters of $M^{\mu }_{\textbf{T} }$ are generically identifiable in the class of trees $\mathcal {T}(r,n,k)$.

Part of our goal in these proofs is to try to give a uniform argument that applies to all equivariant models that contain the Jukes-Cantor as a submodel, to deduce identifiability for these models. A key component of the proof of Corollary 2.7 is to prove that μ has the rank property *RP*(*r*, *k*) and extended rank property *ERP*(*r*, *k*) for the specific values of *r* and *k*. To show this we prove both properties for the Jukes-Cantor model for the particular values of *r* and *k* in the Theorem. Since Jukes-Cantor transition matrices appear in all the other models, and proving these properties requires finding a specific transition matrix, this implies the rank property for the larger models for those values of *r* and *k*. It is probably possible to derive stronger results for the other models with better bounds for *r* by more carefully analyzing the rank property and extended rank property in other models. We discuss this in more detail in Sect. 7.

## Markov Models on Trees

In this section, we review background on the combinatorial structure of trees, including splits and tripartitions associated to tree edges and vertices. We introduce the Markov models on a tree that are the main object of study in this work.

A tree T=(V,E) is a graph that is connected and has no cycles. The degree of a vertex is the number of edges incident to the vertex. A vertex of degree 1 is called a leaf. Phylogenetic trees represent the evolution of taxa with the vertices each representing a different taxa. Taking *T* to be a rooted tree, the closer a vertex is to the root vertex the older that taxa is evolutionarily. The leaves of the tree will represent taxa that are alive today. This gives the leaves special importance since the DNA of those species is observable. The following definitions of phylogenetic trees are standard (see Steel 2016).

### Definition 3.1

Let *T* be a tree and *X* be a set of labels. Let ϕ be a map from *X* to the vertices of *T*. The pair (T,ϕ) is called a *phylogenetic X-tree* if the image of ϕ is exactly the set of leaves of *T* and ϕ is injective.

A *binary phylogenetic X-tree* is a phylogenetic *X*-tree where all non-leaf vertices have degree 3.

A *rooted tree* is a tree with a distinguished vertex called the root.

Removing any edge of a phylogenetic tree separates the leaf labels into two parts, based on which connected component the leaf is in. This leads to following definition.

### Definition 3.2

A *split* *A*|*B* of *X* is a partition of *X* into two nonempty disjoint sets. A split is considered *valid* for an *X*-tree *T* if it is obtained by removing an edge from *T* and taking *A* and *B* to be the two sets of leaf labels for the two component graphs. The set of valid splits of a tree *T* is denoted Σ(T).

Similarly, a tripartition *A*|*B*|*C* of the leaves is valid for a binary tree if it can be obtained by removing an internal vertex and *A* and *B* and *C* are the three sets of leaf labels for the three component graphs.

We call a split *A*|*B* *displayed* for a tree *T* if there is another tree $T^{\prime }$ such that $$A|B \in \Sigma (T') $$ and $$\Sigma (T) \subseteq \Sigma (T'). $$

Note that the notion of a displayed split allows for splits that are not in the tree *T*, but are compatible with that tree. This is useful when we use flattenings to identify tree splits.

Let *T* be a phylogenetic tree. We associate a random variable $X_v$ to each vertex v∈T. Each of the random variables $X_v$ has κ states for a fixed value κ. In general terms, the state space of $X_v$ is $[\kappa ] = \{1,2, \ldots \kappa \}$. If κ=4 this alphabet can be thought of as DNA bases $\{A,C,G,T\}$. There are other values for κ that are relevant biologically, such as κ=20 for amino acids, κ=61 for DNA codons, and κ=2 for DNA where purines and pyrimidines are grouped together. This paper will be primarily focusing on κ=4.

Before defining Markov models on a tree, we will motivate their definition. Consider for each vertex a sequence using the characters of the alphabet. If κ=4 that sequence on the vertex would be the DNA sequence for a gene and each vertex would represent a DNA sequence for a specific taxa. For simplicity, only point substitutions will be considered, i.e. mutations that change one DNA letter to another. Thus each sequence will be of the same length for all vertices in the graph, but possibly have different characters in each location. The Markov model on the tree is a model for how these sequences change over time. If we assume that each site in the sequence evolves independently under the same process, we can describe the model just by assuming we have sequences of length 1, or a single character. Thus, for each vertex we will associate a random variable with κ=4 states where the internal non-leaf vertices will be hidden random variables (since they are unobserved).

To each edge in the graph i→j is associated a Markov transition matrix $M^{ij}$, which is a $\kappa \times \kappa$ matrix whose $(x_i, x_j)$ entry is the conditional probability $P(X_j = x_j | X_i = x_i)$. That is:

$$ M^{ij}(x_i, x_j) = P(X_j = x_j | X_i = x_i). $$

To be a Markov transition matrix $M^{ij}$, we require that all entries are nonnegative, and that the row sums are all equal to one.

Once a Markov transition matrix is specified for each edge, and a distribution of states $\pi \in \Delta _{r-1}$ is specified, we can write down the joint distribution of all states in the model. Let *p*(*x*, *y*) denote the joint probability distribution of all random variables, where *x* corresponds to leaves and *y* corresponds to interior vertices. Let ρ denote the root vertex. Then

$$ p(x, y) = \pi (y_\rho ) \prod _{i \rightarrow j \in E(T)} M^{ij}(x_i,x_j). $$

**Fig. 1:**
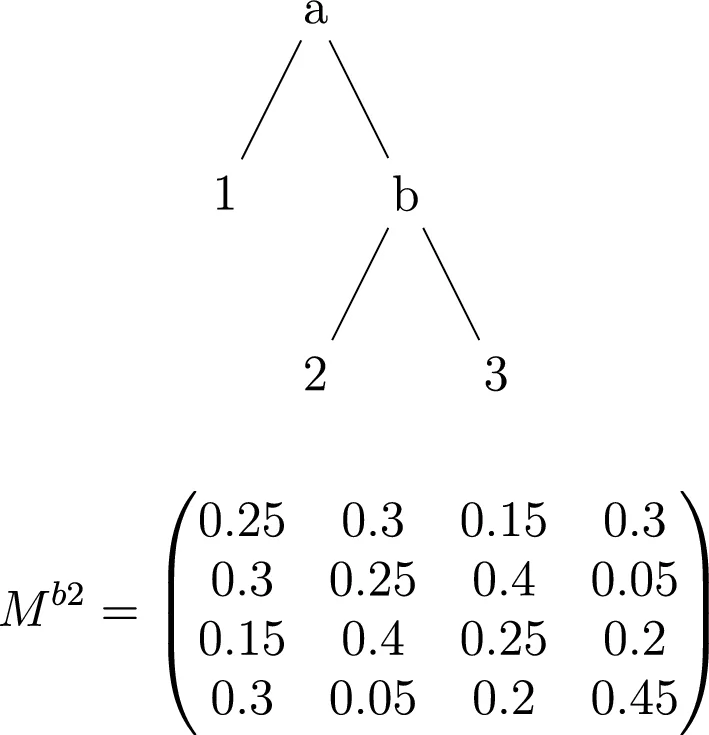
A phylogenetic model with 3 leaves and two internal vertices a and b and a transition matrix

Since we are typically only interested in the distribution of states at the leaves (because we do not have access to data from the taxa at interior nodes), then we have the probability distribution of interest is

$$ p(x) = \sum _{y \in [\kappa ]^{\textrm{Int}(T)}} p(x,y) $$

where $\textrm{Int}(T)$ denotes the interior vertices of *T* (Fig. 1).

### Example 3.3

Consider the rooted tree in Fig. 1. Let the transition matrix written here be associated to the edge from b→2. Label the columns and rows of the matrix with *A*, *C*, *G*, *T*. Then the entries in this transition matrix represent probabilities of DNA substituion when going from the species represented in the vertex *b* to the one represented with the vertex 2. Explicitly the entry $M^{b2}(x_b,x_2)$ is the probability that the variable at $X_b$ mutates from the state $x_b$ to the state $x_2$ at vertex 2. So for example there is a 40% chance $X_b = C$ changes to $X_2 = G$.

Usually when defining a phylogenetic model, we put restrictions on the structure of transition matrices that are used in the model. We call such restriction on the transition matrices a *Markov model* μ . Examples of Markov models are the Jukes-Cantor model, Kimura 2 and 3 parameter models, the Strand Symmetric model, and the general Markov model. The resulting structures on the transition matrices are as follows. The matrix $M^{K3P}$ denotes a generic matrix for the Kimura 3-parameter model:

$$ M^{K3P} = \begin{pmatrix} \alpha & \beta & \gamma & \delta \\ \beta & \alpha & \delta & \gamma \\ \gamma & \delta & \alpha & \beta \\ \delta & \gamma & \beta & \alpha \end{pmatrix}. $$

Note that this matrix only has 3 free parameters, because we require that $\alpha + \beta + \gamma + \delta = 1$. The Kimura 2-parameter model arises as a submodel obtained by requiring that $\beta = \delta$. And the Jukes-Cantor model is a further submodel where $\beta = \gamma = \delta$.

The Strand Symmetric Model is a larger model where the generic transition matrices have the following form:

$$ M^{SSM} = \begin{pmatrix} \alpha & \beta & \gamma & \delta \\ \epsilon & \zeta & \eta & \theta \\ \theta & \eta & \zeta & \epsilon \\ \delta & \gamma & \beta & \alpha \end{pmatrix}. $$

This model has 6 free parameters associated to each transition matrix (again, because the row sums must equal 1). Note that this model contains the JC, K2P, and K3P models as submodels. Finally, the general Markov model makes no restrictions at all on the transition matrices, except that the matrices are transition matrices.

All of these models are examples of what are called *equivariant models*, a useful class of phylogenetic models with key symmetry properties that makes them easier to analyze (Casanellas and Fernández-Sánchez 2025). Suppose that a group *G* acts on the set of states of the random variables. A phylogenetic model is *equivariant* if, for all transition matrices $M_e$ in the model $M_e(x,y) = M_e(g \cdot x, g \cdot y)$, for all *x*, *y* in the state space, and g∈G. That is, $M_e$ should be fixed under simultaneously permuting rows and columns under the action of *G*. Also, we require that the root distribution is fixed under the action of *G*.

Many phylogenetic models are equivariant, including the JC, K2P, K3P, SSM, and GM models. Key features of equivariant models that we will use throughout are:

- The marginalization of an equivariant tree model over some subset of the leaves is a distribution from the equivariant tree model on the restriction to the observed leaves. This also holds for mixture models of equivariant models.
- In an equivariant model, the root can be chosen to be any vertex in the tree without changing the distributions that arise.

A key family of equivariant models are the group-based models, of which the JC, K2P, and K3P are special cases.

### Definition 3.4

Let *G* be a finite abelian group. A phylogenetic model μ is group-based, with respect to the group *G*, if the states of the random variables can be identified with the group elements g∈G and, for all transition matrices *M* in the model, M(g,h)=f(h-g), where $f: G \rightarrow \mathbb {R}$ is some function.

In the next section, we explain how the discrete Fourier transform can simplify the presentation of the group-based models.

## The Discrete Fourier Transform

The discrete Fourier transform is a useful tool that, for certain phylogenetic models, can be applied to simplify the parametrization of the model, to get a parametrization in a new coordinate system that involves polynomials with fewer terms. For the case of group-based models (including the Jukes-Cantor, Kimura 2 and 3 parameter models), it simplifies the phylogenetic model on a tree into a toric variety. For the Strand Symmetric model, the discrete Fourier transform does not produce a toric variety, but another variety that is considerably simpler than the standard parametrization.

### Definition 4.1

Let *G* be a finite abelian group and let $\hat{G} = \textrm{Hom}(G, \mathbb {C}^\times )$ be its dual group of one dimensional representations. Let *f* be a function $f: G \rightarrow \mathbb {C}$. The *discrete Fourier transform* of *f* is the function $\hat{f}: \hat{G} \rightarrow \mathbb {C}$ defined by

$$\begin{aligned} \hat{f}(\chi ) = \sum _{g \in G} f(g)\chi (g). \end{aligned}$$

Definition 4.1 provides the Fourier transform for an arbitrary finite abelian group. We will see how this is applied and can simplify the parametrization for a group-based model and other equivariant models.

First we describe the transformation for the group-based models, and then we show the parametrization for group-based models in Fourier coordinates. Let *T* be a rooted tree with *m* leaves, in a group based model, for each edge we have a transition matrix $M^e$ that satisfies $M^e(g,h) = f^e(g-h)$ for some particular function $f^e: G \rightarrow \mathbb {R}$. Additionally there is a root distribution $\pi : G \rightarrow \mathbb {R}$. For the group-based models we consider here, we will assume that the root distribution π is always the uniform distribution.

Let *H*(*e*) be the head vertex of edge *e* and *T*(*e*) be its tail vertex. Let λ(e) be the set of leaves below *e* and let *Int* be the set of interior vertices of *T*. Then the joint probability of observing $(g_1, \dots , g_m)$ at the leaves is:

$$\begin{aligned} p(g_1 , \dots , g_m)&= \sum _{g \in G^{Int}} \pi (g_r) \prod _{e \in T} M^e(g_{T(e)}, g_{H(e)}) \\&= \sum _{g \in G^{Int}} \pi (g_r) \prod _{e \in T} f^e(g_{T(e)}- g_{H(e)}). \end{aligned}$$

See Chapter 15 of Sullivant (2018) for more details.

Now we compute the Fourier transform $p: G^m \rightarrow \mathbb {R}$ over the group $G^m$. Note that the dual group of a product group is the product of the dual groups. So, as a linear change of coordinates, this transformation is

$$ \hat{p}(\chi _1, \ldots , \chi _m) = \sum _{g_1, \ldots , g_m \in G} \chi _1(g_1) \cdots \chi _m(g_m) p(g_1, \ldots , g_m). $$

The Fourier transform of *p* can be described with the following product formula:

$$ \hat{p}(\chi _1, \dots , \chi _m)= \hat{\pi }\left( \prod _{i=1}^m \chi _i\right) \prod _{e \in T} \hat{f}^e \left( \prod _{i\in \lambda (e)} \chi _i\right) $$

This was first discussed in Evans and Speed (1993) and Hendy and Penny (1996).

### Example 4.2

Consider the rooted 3 leaf claw tree with underlying Jukes-Cantor model. Let $f^i(A)=b_i$ and $f^i(C)=f^i(G)=f^i(T)=a_i$, where we use the index *i* as shorthand for the split $i|\{1,2,3\} \setminus i$. We make the identification that A=(0,0), C=(0,1), G=(1,0), and T=(1,1). The four characters of $\mathbb {Z}_2\times \mathbb {Z}_2$ are the rows of the following character table:

**Table:** 

	A	C	G	T
$\chi _A$	1	1	1	1
$\chi _C$	1	-1	1	-1
$\chi _G$	1	1	-1	-1
$\chi _T$	1	-1	-1	1

So the Fourier transform for a particular edge is

$$ \hat{f}^i(\chi _A) = 3a_i + b_i \quad \quad \text{ and } \quad \quad \hat{f}^i(\chi _C) = \hat{f}^i(\chi _G) = \hat{f}^i(\chi _T) = b_i - a_i. $$

Note that the Fourier transform of the uniform distribution has

$$ \hat{\pi }(\chi _A) = 1, \quad \quad \text{ and } \quad \quad \hat{\pi }^i(\chi _C) = \hat{\pi }^i(\chi _G) = \hat{\pi }^i(\chi _T) = 0 $$

Let $$x_i=3a_i+b_i$$ and $$y_i=b_i- a_i$$.

We use the shorthand $A = \chi _A, \ldots ,$ and we write $q_{AAA} = \hat{p}(\chi _A, \chi _A, \chi _A), \ldots .$ Then we have

$$ q_{AAA} = x_1 x_2 x_3, \quad q_{ACC} = q_{AGG} = q_{ATT} = x_1 y_2 y_3, $$

$$ q_{CAC} = q_{GAG} = q_{TAT} = y_1 x_2 y_3, \quad q_{CCA} = q_{GGA} = q_{TTA} = y_1 y_2 x_3, $$

$$ q_{CGT} = q_{CTG} = q_{GCT} = q_{GTC} = q_{TCG} = q_{TGC} = y_1 y_2 y_3, $$

$$ q_{\chi _1 \chi _2 \chi _3} = 0 \quad \text{ otherwise. } $$

For a group-based model on the group $G = \mathbb {Z}_2 \times \mathbb {Z}_2$, we can express the parametrization in the following form. We have Fourier parameters $a^{A|B}_{g}$ for each split *A*|*B* in the tree, and each group element *g*. The parametrization in Fourier coordinates looks like

$$ q_{g_1 \cdots g_n} = {\left\{ \begin{array}{ll} \prod _{A|B \in \Sigma (T)} a^{A|B}_{\sum _{a \in A} g_a} & \text{ if } \sum _{i = 1}^n g_i = (0,0) \\ 0 & \text{ otherwise. } \end{array}\right. } $$

Then, depending on the specific model, there might be equalities that hold between some the Fourier parameters. In the Jukes-Cantor model, for each split $A|B \in \Sigma (T)$, we have that

$$ a^{A|B}_{(0,1)} = a^{A|B}_{(1,0)} = a^{A|B}_{(1,1)}. $$

Note that the Splits Equivalence Theorem (see Steel 2016) gives a correspondance between valid splits for a tree and edges in the tree. So we will sometimes just use parameters $a^e_g$, where *e* is an edge label, when this is appropriate.

The Fourier transform also applies to other group-based models besides the 4-state models. For instance, the *fully symmetric model* which is a generalization of the Jukes-Cantor model to arbitrary number κ of states (where all off-diagonal entries are equal, and all diagonal entries are equal), can be diagonalized using the Fourier transform over the group $\mathbb {Z}_\kappa$.

## Tensors and Flattenings

In this section, we discuss how to think about the probability distribution associated to a phylogenetic mixture model as a multi-way tensor. The tensor perspective is useful, because we can also consider flattenings of our tensor to lower order tensors, and matrices, in order to use Kruskal’s theorem and matrix ranks of flattenings to prove identifiability results. The approach of using Kruskal’s theorem to prove identifiability results first appeared in Allman et al. (2009).

For a tree with *n* leaves, and random variables with κ states, the joint probability distribution in our model is naturally considered as a $\kappa \times \dots \times \kappa$ *n*-tensor, inside of $\bigotimes _{i = 1}^n \mathbb {R}^\kappa$. From this order-*n* tensor, we can also consider the tensor naturally as a lower order tensor, by grouping indices according to a set partition of the coordinates. Such a reorganization of the tensor is called a *flattening*.

### Definition 5.1

Let *P* be an order-*n* tensor in $\bigotimes _{i =1}^n \mathbb {R}^{\kappa _i}$. Let $A_1| \dots |A_k$ be a partition of [*n*]. The flattening of *P* according to $A_1| \dots |A_k$, is an order-*k* tensor obtained by grouping indices according to the partition $A_1| \dots |A_k$, and is denoted $\textrm{Flat}_{A_1| \cdots | A_k}(P)$. Specifically, $\textrm{Flat}_{A_1| \cdots | A_k}(P)$ is the image of *P* under the natural map

$$ \bigotimes _{i =1}^n \mathbb {R}^{\kappa _i} \rightarrow \bigotimes _{i = 1}^k \left( \bigotimes _{a \in A_i} \mathbb {R}^{a} \right) . $$

In particular, $\textrm{Flat}_{A_1| \cdots | A_k}(P)$ is a $\prod _{a_1 \in A_1} \kappa _{a_1} \times \cdots \times \prod _{a_k \in A_1} \kappa _{a_k}$ tensor.

Note that in the specific case when $\kappa _i = \kappa$ for all *i*, and if k=2, then we have our partition *A*|*B* and $\textrm{Flat}_{A|B}(P)$ is a matrix of size $\kappa ^{\#A} \times \kappa ^{\#B}$.

### Example 5.2

Let *T* be a $3\times 3 \times 3$ tensor with entries $t_{ijk}$, and consider the partition A|B=1|23. Then

$$ \textrm{Flat}_{1|23}(T) =\begin{pmatrix} t_{111} & t_{112} & t_{113} & t_{121} & t_{122} & t_{123} & t_{131} & t_{132} & t_{133} \\ t_{211} & t_{212} & t_{213} & t_{221} & t_{222} & t_{223} & t_{231} & t_{232} & t_{233} \\ t_{311} & t_{312} & t_{313} & t_{321} & t_{322} & t_{323} & t_{331} & t_{332} & t_{333} \\ \end{pmatrix} $$

One key advantage to considering our probability distributions as tensors is that we can use tensor rank as a tool to get at questions of identifiability in phylogenetic models. To explain the key tool in this area, Kruskal’s theorem, we need to review some concepts related to tensor rank.

For j=1,2,3, let $\boldsymbol{m}^j = (m^j(1), \ldots , m^j(\kappa _j)) \in \mathbb {R}^{\kappa _j}$. Let $\boldsymbol{m}^1 \otimes \boldsymbol{m}^2 \otimes \boldsymbol{m}^3$ denote the $\kappa _1 \times \kappa _2 \times \kappa _3$ tensor whose (*u*, *v*, *w*) entry is $m^1(u)m^2(v) m^3(w)$. The tensor $\boldsymbol{m}^1 \otimes \boldsymbol{m}^2 \otimes \boldsymbol{m}^3$ is called a *rank 1 tensor*. A tensor is said to have rank *r* if it can be written as the sum of *r* rank 1 tensors, and cannot be written as the sum of r-1 rank 1 tensors. Kruskal’s theorem concerns the uniqueness of the representation of a rank *r* tensor as the sum of *r* rank 1 tensors. To express this, we first introduce the triple product notation of matrices.

### Definition 5.3

Let $M_j$ be a $r \times \kappa _j$ matrix with *i*th row $\boldsymbol{m_i^j} = (m_i^j(1), \dots m_i^j(\kappa _j)).$ Then let $[M_1, M_2, M_3]$ be defined by

$$ [M_1, M_2, M_3] = \sum _{i=1}^r \boldsymbol{m_i^1} \otimes \boldsymbol{m_i^2} \otimes \boldsymbol{m_i^3}. $$

The triple product $[M_1, M_2, M_3]$ is a $\kappa _1 \times \kappa _2 \times \kappa _3$ tensor and its (*u*, *v*, *w*) entry is

$$ [M_1, M_2, M_3]_{(u,v,w)} = \sum _{i=1}^r m_i^1(u) m_i^2(v) m_i^3(w). $$

Note that there is a natural nonuniqueness that is always present. If Π is an r×r permutation matrix, and $D_1, D_2, D_3$ are r×r diagonal matrices such that $D_1D_2D_3 = Id_r$, then

$$ [M_1, M_2, M_3] = [\Pi D_1 M_1, \Pi D_2 M_2, \Pi D_3 M_3]. $$

If

$$ [M_1, M_2, M_3] = [N_1, N_2, N_3] $$

and there exist Π and $D_1, D_2, D_3$ such that

$$ [N_1, N_2, N_3] = [\Pi D_1 M_1, \Pi D_2 M_2, \Pi D_3 M_3] $$

then we say that $$N_1, N_2, N_3$$ are related to $$M_1, M_2, M_3$$ by scaling and simultaneous permutation of the rows. If this is the only way we can have $$[M_1, M_2, M_3] = [N_1, N_2, N_3]$$ then we say that $$[M_1, M_2, M_3]$$ uniquely determines $$M_1$$, $$ M_2$$, $$M_3$$ up to simultaneous permutation and scaling of the rows. Note that this means that the rank one tensors that appear in the decomposition are themselves unique.

The existence of a unique decomposition in the Kruskal theorem depends on the notion of the Kruskal rank of a matrix.

### Definition 5.4

Let *M* be a $r \times \kappa$ matrix. The *Kruskal rank of a matrix M*, written as $\textrm{rank}_{K}(M)$ is the largest *k* such that every subset of *k* rows of *M* is linearly independent.

Note that $\textrm{rank}_{K}(M) \le \textrm{rank}(M)$ but if *M* is of full row rank (that is, $\textrm{rank}(M) = r$), it will also be full Kruskal rank. Now we are ready to state Kruskal’s Theorem.

### Theorem 5.5

(Kruskal’s Theorem) (Kruskal 1977) For j=1,2,3, let $M_j$ be an $r \times \kappa _j$ matrix and let $I_j= \textrm{rank}_K M_j$. If

$$ I_1 +I_2 +I_3 \ge 2r +2 $$

then $[M_1, M_2, M_3]$ uniquely determines $M_1$, $M_2$, and $M_3$ up to simultaneous permutation and scaling of the rows.

The motivation for considering tensor rank comes from the fact that the probability distribution from a single tree can be represented as a triple product, when considered around a given vertex. Specifically, let *T* be a tree, let *v* be a trivalent vertex in *T*. We can assume that *v* is the root of the tree. The vertex *v* induces a tripartition *A*|*B*|*C* of the leaves. Let $\textrm{diag}(\pi )$ be a diagonal matrix with the root distribution on the diagonal. Let $M_A$ be the $\kappa \times \kappa ^{\#A}$ matrix which is the flattenings of the conditional distribution of $X_A$ given $X_v$. Similarly, define $M_B$ and $\tilde{M}_C$ as conditional distributions of $X_B$ given $X_v$ and $X_C$ given $X_v$, respectively. Then, if *P* is the joint distribution of states at the leaves of our tree *T*, we have

$$ \textrm{Flat}_{A|B|C}(P) = [M_A, M_B, \textrm{diag}(\pi ) \tilde{M}_C] =[M_A, M_B, M_C]. $$

where we let $M_C = \textrm{diag}(\pi ) \tilde{M}_C.$ This representation was first developed in Allman et al. (2009), and used to prove identifiability results in a number of hidden variable models including phylogenetic models.

Note that we could have absorbed the diagonal matrix $\textrm{diag}(\pi )$ into any of $M_A, M_B$ or $\tilde{M}_C$. Since we are always working with generic distributions, this would not change the Kruskal rank or tensor rank. Hence we can consider $M_A$, $M_B$, and $M_C$ as either conditional distributions given $X_v$, or just as joint distributions.

## A General Theorem on Identifiability of Phylogenetic Mixtures

Our goal in this section is to prove our main general result about the identifiability of phylogenetic mixture models, Theorem 2.6. To do this, we must first define the rank property, extended rank property, the standard Markov model property, and the No Shuffling Property, and show how they are related to identifiability of mixture models.

### Definition 6.1

The Markov model μ is said to have the *Rank property* *RP*(*r*, *n*) if the following holds: For any trees $T_1, \ldots , T_r$ on *n* leaves, and generic probability distributions $P_i \in M^\mu _{T_i}$, the matrix

$$ \begin{pmatrix} \textrm{Flat}_{[n-1]|n}(P_1)&\textrm{Flat}_{[n-1]|n}(P_2)&\cdots&\textrm{Flat}_{[n-1]|n}(P_r) \end{pmatrix} $$

has rank rκ.

### Definition 6.2

The Markov model μ is said to have the *extended rank property* *ERP*(*r*, *n*) if the following holds: For any tree $T_1$ on n+1 leaves that does not contain the split $[n-1]| \{n, n+1\}$, and any trees $T_2, \ldots T_r$ on *n* leaves, and generic probability distributions $P_i \in M^\mu _{T_i}$, the matrix

$$ \begin{pmatrix} \textrm{Flat}_{[n-1]|\{n, n+1\}}(P_1)&\textrm{Flat}_{[n-1]|n}(P_2)&\cdots&\textrm{Flat}_{[n-1]|n}(P_r) \end{pmatrix} $$

has rank $$(r-1) \kappa + \kappa ^2$$.

Note that in both the rank property and extended rank property, the rank of the resulting matrix will achieve its maximum possible value, (since the rank is equal to the number of columns). Before proving statemnts about the rank property and extended rank property, we give a quick remark about how marginalization interacts with flattenings.

### Remark 6.3

Let $$P \in \bigotimes _{i = 1}^m \mathbb {R}^\kappa $$ be a probability distribution. Let *A*|*B* be a partition of [*m*]. Let $A^{\prime }\subseteq A$ and let $B^{\prime }\subseteq B$. We can compute the marginalization of *P* on the set of variables indexed by $A^{\prime }\cup B^{\prime }$. Let $P^{\prime }$ denote the associated probability distribution. Then $$\textrm{Flat}_{A' | B'}(P')$$ is obtained from $$\textrm{Flat}_{A | B}(P)$$ by performing row sums and column sums. In particular, marginalization corresponds to left-multiplication by a row-summation matrix and right-multiplication by a column summation matrix on the level of flattenings. In particular, rank of the flattening can only decrease (or stay the same) under marginalization.

A useful fact about both the rank property and extended rank property is that they satisfy persistence properties in their values.

### Proposition 6.4

Let μ be a Markov model such that the rank property *RP*(*r*, *n*) holds. Then the rank property *RP*(*s*, *m*) holds for all s≤r and m≥n. Similarly, if the extended rank property *ERP*(*r*, *n*) holds, then the extended rank property *ERP*(*s*, *m*) holds for all s≤r and m≥n.

### Proof

Suppose that *RP*(*r*, *n*) holds. Let

$$ Q = \begin{pmatrix} \textrm{Flat}_{[n-1]|n}(P_1)&\textrm{Flat}_{[n-1]|n}(P_2)&\cdots&\textrm{Flat}_{[n-1]|n}(P_r) \end{pmatrix}. $$

Then the associated matrix $Q^{\prime }$ which is used to check *RP*(*s*, *n*) for s≤r is obtained from *Q* by taking a submatrix of the columns of *Q*. Since *Q* had full column rank $Q^{\prime }$ must have full column rank as well.

Now suppose that m≥n. Construct the matrix $Q^{\prime }$ to check *RP*(*r*, *m*). Each of the distributions obtained to form *Q* is gotten by marginalizing the corresponding distribution from $Q^{\prime }$. We can assume we are marginalizing some collection of random variables besides the last variable, *m*. On the level of matrices, *Q* is obtained from $Q^{\prime }$ by adding together rows of $Q^{\prime }$. Since we assumed that *Q* has rank rκ and *Q* is obtained as a linear transformation of $Q^{\prime }$, and has exactly rκ columns, it must also have had rank rκ, so μ satisfies *RP*(*r*, *m*).

The same arguments work verbatim for the extended rank property. □

One further property we need of our phylogenetic models is a certain notion of standardness of the set of transition matrices, as that will allow us to deduce that the matrices have a suitable Kruskal rank.

### Definition 6.5

A Markov model μ is *standard* if it satisfies the following conditions:

- the model is generically identifiable on trees
- the model contains the identity matrix, and
- for any two generic transition matrices $M_1$ and $M_2$ in the model, any pair of column vectors obtained from taking two columns of $M_1$, or one column of $M_1$ and one column of $M_2$ are linearly independent.

### Proposition 6.6

Let μ be a standard Markov model. Let $T_1, \ldots , T_r$ be trees on *n* leaves with n≥2. Let $P_1, \ldots , P_r$ be generic probability distributions $P_i \in M^\mu _{T_i}$. Then the matrix

$$ Q = \begin{pmatrix} \textrm{Flat}_{[n-1]|n}(P_1)&\textrm{Flat}_{[n-1]|n}(P_2)&\cdots&\textrm{Flat}_{[n-1]|n}(P_r) \end{pmatrix} $$

has Kruskal rank ≥2 for any *r*.

### Proof

By marginalizing, we can assume that all the trees are just a single edge with 2 leaves. To see that the matrices have Kruskal rank ≥2, we just need to look at the case where r=1,2, and taking any pair of columns from the resulting two transition matrices. However, the definition of standard specifically covers these cases, which shows that matrix *Q* has Kruskal rank at least 2. □

Last we come to the No Shuffling Property, which says that parts of probability distributions on different trees cannot be combined to get a distribution on another tree.

### Definition 6.7

A Markov model μ is said to the have the *No Shuffling Property* if the following holds: For any trees $T_1, \ldots , T_r$ with n≥3 leaves and generic probability distributions $P_i \in M^\mu _{T_i}$, let *Q* be the matrix

$$ Q = \begin{pmatrix} \pi _1 \textrm{Flat}_{[n-1]|n}(P_1)&\pi _2 \textrm{Flat}_{[n-1]|n}(P_2)&\cdots&\pi _r\textrm{Flat}_{[n-1]|n}(P_r) \end{pmatrix} $$

where $$\pi \in \Delta _r$$ is generic. Form a new matrix $Q^{\prime }$ by taking κ not necessarily distinct columns of *Q*, and let *P* be the resulting tensor. If $$P \in M^\mu _T$$ for some *T*, then all columns of $Q^{\prime }$ must have come from the same $$P_i.$$

The importance of the No Shuffling Property is that it implies that if we permute the columns of *Q*, we can still tell which columns belong together (coming from the same distribution and same tree). This follows because it is not possible to get a distribution from a tree by taking a combination of some of the columns from different trees.

Our goal at this point is to show how a model that satisfies these properties can be made to follow the proof strategy from Rhodes and Sullivant (2012), closely following the outline of those ideas, but substituting in these new properties in place of directly using the General Markov model to prove the results.

### Lemma 6.8

Let μ be a standard Markov model that satisfies the extended rank property *ERP*(*r*, *k*) and let $r \le \kappa ^{k -1}$. Let *P* be a probability distribution that is a generic mixture of *r* generic distributions from trees $T_1, \ldots , T_r$ on *n* leaves. Suppose that *A*|*B* is a split with #A>k and #B>k.

1. If every tree $T_1, \ldots , T_r$ displays the split *A*|*B* then $\textrm{rank}(\textrm{Flat}_{A|B}(P)) \le \kappa r.$
2. If there is some tree $T_i$ that does not display the split *A*|*B* then $\textrm{rank}(\textrm{Flat}_{A|B}(P)) > \kappa r.$

### Proof

Suppose *A*|*B* is displayed in all trees in $\textbf{T} = (T_1, \ldots , T_r).$ By the definition of displayed, there must exist binary trees $T_1', \ldots , T_r'$ that contain the split *A*|*B* and the splits $\Sigma (T_1), \ldots , \Sigma (T_r)$, respectively. Since we can get back to the trees $T_1, \ldots , T_r$ by setting transition matrices along some edges equal to the identity, it suffices to assume that all the $T_1, \ldots , T_r$ are binary. Let *A*|*B* be associated to the edge $e_i = (a_i, b_i)$ in $T_i.$ Then one sees that

$$ \textrm{Flat}_{A|B}(P) = M^T_A Q M_B. $$

Here *Q* is the $\kappa r \times \kappa r$ block-diagonal matrix whose *i*th $\kappa \times \kappa$ block gives the joint probability distribution of states for the random variables at $a_i$ and $b_i$, weighted by the component proportion $\pi _i.$ The matrices $M_A$, $M_B$ are stochastic, of sizes $\kappa r \times \kappa ^{\# A}$, $\kappa r \times \kappa ^{\# B}$, with entries in the *i*th block of κ rows giving probabilities of states of variables in *A*, *B* conditioned on states at $a_i$, $b_i$. Since *Q* is a $\kappa r \times \kappa r$ matrix it has rank at most κr, which implies that $\textrm{Flat}_{A|B}(P)$ has rank at most κr as well.

Suppose next that *A*|*B* is not compatible with at least one of the trees in $\textbf{T},$ say $T_1.$ To show that $\text {Flat}_{A|B}(P)$ generically has rank greater than κr, it is enough to give a single choice of parameters producing such a rank. Indeed, this follows from Proposition 3.2 in Rhodes and Sullivant (2012), applied to the model and the variety of matrices of rank at most κr.

For each $T_i$ with i>1 choose all Markov matrices for all internal edges of $T_i$ to be the identity, $I_{\kappa }$. Since $T_1$ is not compatible with *A*|*B*, by Theorem 3.8.6 of [19], it has an edge e=(c,d), with associated split *C*|*D*, such that all four sets A∩C, A∩D, B∩C, B∩D are nonempty. For all internal edges of $T_1$ except *e*, choose Markov matrices to be $I_{\kappa }$ as well. Since the effect of an identity matrix on an edge is the same as contracting that edge. For simplicity, we will refer to these contracted trees by their original labeling and just work with these contracted edge trees. This reduces our model to the case where for i>1, $T_i$ is a star tree with central node $a_i$, and $T_1$ has the form of two star trees, on *C* and on *D*, that are joined at their central nodes by *e*.

Let $$P= P_1 + P'$$ where $$P_1$$ is the mixture component from $$T_1$$, and $P^{\prime }$ the sum of the components on the star trees $$T_2= \dots = T_r$$. Then one sees that

$$ M_2:= Flat_{A|B}(P') = N^T_A R N_B, $$

with *R* an $\kappa (r-1) \times \kappa (r-1)$ diagonal matrix giving the distribution of states at $a_i$ in components 2,…,r weighted by $\pi _i$ and $N_A$, $N_B$ are stochastic matrices of sizes $\kappa (r-1) \times \kappa ^{\# A}$, $\kappa (r-1) \times \kappa ^{\# B}$ with entries giving conditional probabilities of states of variables in *A*, *B* conditioned on states/components at the $a_i$. By choosing the positive root distributions at the nodes $a_i$, and positive $\pi _i$, *R* is ensured to have positive diagonal entries, and hence have full rank.

Consider $P_1$ where all matrices on pendant edges of $T_1$ are chosen to be $I_{\kappa }$. Also, both the root distribution at *c* and $M_e$ are chosen to have all positive entries. Then let

$$ M_1:= \textrm{Flat}_{A|B}(P_1) = N^T_{1,A} R_1 N_{1,B}. $$

where $R_1$ is a $\kappa ^2 \times \kappa ^2$ diagonal matrix with entries giving the joint distribution at *c* and *d* weighted by $\pi _1$, and $N_{1,A}$, $N_{1,B}$ have all zero entries except for a single 1 in each row, and full row rank. Thus $M_1$ has rank $\kappa ^2$. Moreover, it has at most one non-zero entry in each row and column, so both $\textrm{im}(M_1)$ and $\ker (M_1)$ are coordinate subspaces.

Note that the matrices $( N_{1,A}, N_A)$ and $( N_{1,B}, N_B)$ are matrices of the form in the definition of the rank property, so each of these matrices has rank $\kappa ^2 + \kappa (r-1)$, since μ has the extended rank property. Furthermore, the two factorizations for $M_1$ and $M_2$, mean we can write

$$ \textrm{Flat}_{A|B}(P)= M_1 + M_2 = (N_{1,A}^T \, \, N_A^T) \begin{pmatrix}R_1 & 0 \\ 0 & R \end{pmatrix} \begin{pmatrix} N_{1,B} \\ N_B \end{pmatrix}. $$

Since all the matrices in the product have maximal rank, we see that $\text {rank}(M_1+M_2)> \kappa r$ and thus we are done. □

The next goal is to show how to use the ranks of flattenings from Lemma 6.8 to identify the key features of a phylogenetic mixture model.

### Lemma 6.9

Suppose that $\textbf{T} = (T_1, \ldots , T_r) \in \mathcal {T}(r,n,k)$, and let $P \in M^\mu _{\textbf{T}}$ be generic. Suppose that μ satisfies *RP*(*n*, *k*) and *ERP*(*n*, *k*). Then we can use ranks of flattenings to find a partition on [*n*] into three sets *A*, *B*, *C* with $\#A \ge k$, $\#B \ge k$, such that A|B∪C and B|A∪C are valid splits for all the trees $T_1, \ldots , T_r$.

### Proof

To find such an *A*|*B*|*C*, we simply test all the tripartitions that have $$\#A \ge k$$ and $$\#B \ge k$$. We know, by the definition of $$\mathcal {T}(r,n,k)$$, that there must exist a triple $A^{\prime }\left|B^{\prime }\right|C^{\prime }$ where each of the splits $A^{\prime }|B^{\prime }\cup C^{\prime }$, $B^{\prime }|A^{\prime }\cup C^{\prime }$, and $C^{\prime }|A^{\prime }\cup B^{\prime }$ holds for all the trees $$T_1, \ldots , T_r$$. According to Lemma 6.8, a split *D*|*E* has $$\textrm{rank}\, \textrm{Flat}_{D|E}(P) \le r\kappa $$ if and only if the split *D*|*E* appears in all the trees $$T_1, \ldots , T_r$$, provided that $$\kappa ^{\#D} > r \kappa $$ and $$\kappa ^{\#E} > r \kappa $$. By our assumptions on *k*, this holds. So, the ranks of flattenings will find a triparition *A*|*B*|*C* of the desired type. □

Note that just because we find A|B∪C and B|A∪C using the ranks of flattenings, it does not necessarily imply that C|A∪B is a valid split in all the trees. This is in spite of the fact that we know that there exists a triple $A^{\prime }\left|B^{\prime }\right|C^{\prime }$ that is a common tripartition to all trees.

### Example 6.10

Consider two caterpillar trees $T_1$ and $T_2$ with nontrivial splits

$$ \{12|34567, 123|4567, 1234|567, 12345|67 \} \text{ and } \{12|34567, 124|3567, 1234|567, 12345|67 \}. $$

Both trees have a common tripartition, 1234|5|67. However, if we only found the splits 12|34567 and 12345|67, which are common to both trees, we cannot conclude that 345|1267 is also in both trees.

Flattenings cannot necessarily find that valid tripartition alone. However, we can use Lemma 6.9 to prove our identifiability results anyways. This result follows the proof of Theorem 4.4 of Rhodes and Sullivant (2012).

### Lemma 6.11

Let μ be a standard Markov model that satisfies *RP*(*r*, *k*) and the No Shuffling Property. Suppose that the trees $\textbf{T} = (T_1, \ldots , T_r)$ have a known common tripartition *A*|*B*|*C* with $\#A \ge \#B \ge k$. Then both $\textbf{T}$ and the numerical parameters of the μ-mixture model on $\textbf{T}$ are generically identifiable.

### Proof

Since all the trees in $\textbf{T}$ share a common tripartition, we can write a distribution in the mixture model as a triple product

$$ \textrm{Flat}_{A|B|C}(P) = [M_A, M_B, M_C] $$

where the matrices are $M_A$, $M_B$, and $M_C$ are as described at the end of Sect. 5.

Since the model μ satisfies the rank property *RP*(*r*, *k*), for generic choices of parameter the matrices $M_A$ and $M_B$ will each have rank rκ. The fact that μ is standard guarantees that $M_C$ has Kruskal rank ≥2.

The triple product representation expresses $\textrm{Flat}_{A|B|C}(P)$ as the sum of rκ rank 1 tensors. But since

$$ \textrm{rank}_K(M_A) + \textrm{rank}_{K}(M_B) + \textrm{rank}_K(M_C) \ge r \kappa + r \kappa + 2 = 2r\kappa + 2 $$

we can apply Kruskal’s theorem to see that the matrices $M_A$, $M_B$, and $M_C$ can be recovered up to scaling and permuting the rows.

Each of the rows of the recovered matrices $M_A$, $M_B$, $M_C$ will have entries from a scaled slice from a tree distribution on a subtree of one of the $T_i$ (the subtree from the common vertex to the leaves *A*, *B* or *C*, respectively). We need to group these rows together by the mixture components they come from. However, since we have assumed that μ satisfies the No Shuffling Property, there is only one way to do this for $M_A$. Since the ordering of rows of $M_A$ denotes the order of the rows of $M_B$ and $M_C$, we can reassemble each scaled probability distribution $\pi _i P_i$ as the triple product $[M_{i,A}, M_{i,B}, M_{i,C}]$ for submatrices of $M_A, M_B, M_C$ respectively.

From the scaled distribution $\pi _i P_i$ we recover the mixing weight via the sum

$$ \pi _i = \sum _{(j_1, \ldots , j_n) \in [\kappa ]^n} \pi _i P_i(j_1, \ldots , j_n). $$

Then we can get the distribution $P_i$ for the single tree $T_i$. We use that μ is a standard model, so the tree parameter $T_i$ and numerical parameters are generically identifiable. □

The next Lemma closely follows the proof of Theorem 4.6 in Rhodes and Sullivant (2012).

### Lemma 6.12

Let μ be a standard Markov model that satisfies *RP*(*r*, *k*) and the No Shuffling Property. Suppose that the trees $\textbf{T} = (T_1, \ldots , T_r)$ have a known tripartition with *A*|*B*|*C* with $\#A \ge \#B \ge k$ such that A|B∪C and B|A∪C are valid splits in all trees $T_1, \ldots , T_r$. Then both $\textbf{T}$ and the numerical parameters of the μ-mixture model on $\textbf{T}$ are generically identifiable.

### Proof

Let *P* be a generic distribution of the μ mixture model. Fix some c∈C, let $D(c) = A \cup B \cup \{c\}$. Consider the marginalization of *P* to the set *D*(*c*), and call this distribution $P_c$. This is the probability tensor for the induced *r*-tuple of trees

$$ \textbf{T}|_{D(c)} = (T_1|_{D(c)}, \ldots , T_r|_{D(c)}). $$

Note that all the trees of $\textbf{T}|_{D(c)}$ share the common triparition $A|B|\{c\}$, which satisfies the conditions of Lemma 6.11, so that tree parameters and numerical parameters are generically identifiable on this subset of the leaves. We also have that

$$ \textrm{Flat}_{A|B|\{c\} }(P_c) = [M_A, M_B, M_c]. $$

Since μ satisfies the rank property *RP*(*r*, *k*), we can assume that $M_A$ has full rank. Thus, there is a right inverse matrix $Q_A$ with the property that $M_A Q_A = I_{r \kappa }$, where $I_{r \kappa }$ is the rκ identity matrix. Our goal is to use the matrix $Q_A$ to finish the disentangling of distributions that go into *P*.

Now, since A|B∪C is a valid split in all the trees $\textbf{T}$, we can factor

$$ \textrm{Flat}_{B \cup C | A}(P) = M^T_{B \cup C} \Pi \widetilde{M_A} $$

where $\widetilde{M_A}$ and $M_{B \cup C}$ are stochastic matrices of probabilities of the states of the leaves in *A* and B∪C conditioned on which tree $T_i$ that portion of the matrix corresponds to and on the states at the root $w_i$ in each tree where $T_i|_A$ attaches to the rest of $T_i$. The matrix Π is a diagonal matrix whose entries are the root distribution probabilities times the mixing weights. A key feature is that the components can be ordered so that all the parameters associated to a particular tree $T_i$ can be assumed to be in the same block of rows.

Now we can write $M_A = R\widetilde{M_A}$ where *R* is a block diagonal matrix whose *i*th block gives the conditional probability of state changes from the root $w_i$ to the adjacent vertex in $T_i$ away from *A*. We can assume that this matrix is invertible since the blocks are just μ-transition matrices, and generically those are nonsingular since μ is standard.

Now we can compute

$$ \textrm{Flat}_{B\cup C|A}(P) Q_A = M^T_{B \cup C} \Pi R^{-1} M_A Q_A = M^T_{B\cup C} \Pi R^{-1}. $$

This shows that taking the columns of $\textrm{Flat}_{B \cup C | A}(P) Q_A$ in blocks of κ we obtain entries associated to only one mixture component at a time. Multiplying a block of those columns by the associated corresponding rows of $M_A = R \widetilde{M_A}$ we obtain a single mixture component $\pi _i P_i$ from the single tree $T_i$, multiplied by the mixing weight $\pi _i$. We can identify $\pi _i$ by summing all entries of this tensor, as in the proof of Lemma 6.11. Then the fact that the tree and numerical parameters are generically identified for the model μ for a single tree completes the proof. □

Now we are in a position to combine all components to complete the proof of the main structural theory on identifiability of mixture models.

### Proof of Theorem 2.6

Let *P* be a generic distribution from a mixture model with some $\textbf{T} = (T_1, \ldots , T_r) \in \mathcal {T}( r,n,k)$. We need to show that from *P* alone, we can find $\textbf{T}$ and the numerical parameters of the model. According to Lemma 6.9, it is possible to use ranks of flattenings to find a tripartiion *A*|*B*|*C* of the leaf set such that A|B∪C and B|A∪C are valid splits in all the trees $T_i$, and both $\#A \ge k$ and $\#B \ge k$. Once those are identified, Lemma 6.12 shows that it is possible to identify the trees $T_i$, the mixing weights, and the numerical parameters for each tree. □

## Rank property, Extended Rank Property, and Standard Property for the Jukes-Cantor Model

In this section, we will prove that the Jukes-Cantor model satisfies the Rank Property, Extended Rank Property, and Standard condition with appropriate conditions on *r* and *k*. While this might seem narrow, a key observation is that if any of these properties are satisfied for a certain model μ, they are also satisfied for all models $\mu^{\prime }$ that contain μ as submodels. Since the Jukes-Cantor model is contained in all the equivariant models as a submodel, this will prove that those three properties are also satisfied for those models.

First we will prove that the models under consideration in this paper are all standard models, the most straightforward property to prove for a model.

### Lemma 7.1

All equivariant models are standard Markov models. In particular, the JC, K2P, K3P, SSM, and GMM models are all standard Markov models.

### Proof

All these models contain the identity matrix, and are known to be identifiable on trees (e.g. using the tensor rank arguments from Allman et al. (2011) or Casanellas and Fernández-Sánchez (2025) for the more general result on equivariant models. ).

Equivariant models give a set of transition matrices that is a linear space. Since such a set is an irreducible variety, it suffices to prove the property on pairs of matrices giving the correct Kruskal rank on any two specific matrices in the model. Recall that the *fully symmetric model* consists of all transition matrices that have one value *b* for all off diagonal entries and a different value *a* for all diagonal entries. Since the fully symmetric model is a subset of any equivariant model, it then suffices to prove the result for the fully symmetric model.

In the fully symmetric model, we can take the identity matrix, together with any other matrix in the model that does not have rank 1 (e.g.  take b=ϵ and $a = 1 - (\kappa - 1) \epsilon$). This pair of matrices will satisfy the condition on independence of column vectors. □

A key fact that we will use (and that was also used in the proofs in Rhodes and Sullivant (2012)), is that matrices that are generalized Vandermonde matrices have full rank for generic choices of parameters.

### Definition 7.2

Let $x^{u_1}, \ldots , x^{u_r}$ be monomials in $\mathbb {C}[x_1, \ldots , x_n]$. Let $v_1, \ldots , v_s \in \mathbb {C}^n$ be vectors. Then the matrix

$$ V(u_1, \ldots , u_r; v_1, \ldots , v_s) = \begin{pmatrix} v_1^{u_1} & v_1^{u_2} & \cdots & v_1^{u_r} \\ \vdots & \vdots & \ddots & \vdots \\ v_s^{u_1} & v_s^{u_2} & \ddots & v_s^{u_r} \end{pmatrix} $$

is called the *generalized Vandermonde matrix*.

### Proposition 7.3

Let $x^{u_1}, \ldots , x^{u_r}$ be distinct monomials in $\mathbb {C}[x_1, \ldots , x_n]$. Let $v_1, \ldots , v_r \in \mathbb {C}^n$ be generic vectors. Then the generalized Vandermonde matrix $V(u_1, \ldots , u_r; v_1, \ldots , v_r)$ has rank *r*.

### Proof

Since all vectors $u_1, \ldots , u_r$ are distinct, there exists an integer vector $d = (d_1, \ldots , d_n)$, such that the integers $u_1^Td, \ldots , u_r^Td$ are all distinct. The resulting univariate Vandermonde matrix $V(u_1^Td, \ldots , u_r^Td; t_1, \ldots , t_r)$ will be invertible for generic choices of $t_1, \ldots , t_r$. However, this univariate Vandermonde matrix arises as a specialization of the generalized Vandermonde matrix $V(u_1, \ldots , u_r; v_1, \ldots , v_r)$ where we take $v_i = ( t_i^{d_1}, \ldots , t_i^{d_n})$. Since this specialization had full rank, the generalized Vandermonde matrix has full rank for generic choices of $v_1, \ldots , v_r \in \mathbb {C}^n$. □

More generally, we have the following useful fact about the matroid that is determined by a generalized Vandermonde matrix.

### Proposition 7.4

Let $x^{u_1}, \ldots , x^{u_r}$ be not necessarily distinct monomials in $\mathbb {C}[x_1, \ldots , x_n]$. Suppose that there are *l* distinct monomials among them. Let $v_1, \ldots , v_s \in \mathbb {C}^n$ be generic vectors. Then any nonzero vector in the row span of $V(u_1, \ldots , u_r; v_1, \ldots , v_s)$ has at least l-s+1 nonzero entries.

### Proof

We can assume that there are no repeats in the list of monomials, since any repeats must necessarily yield repeated nonzero entries of vectors in the row span. Suppose that there is a nonzero vector *x* in the row span of $$V = V(u_1, \ldots , u_r; v_1, \ldots , v_s)$$ that had *s* or more zero entries. Let *S* be a set of exactly *s* of those entries. After permuting columns, we can assume that those are the first *s* entries of *x*. The fact that *x* is a nonzero vector of the row span means that there is an invertible s×s matrix *M* such that *MV* has *x* as the bottom row. Consider the square-submatrix $$V' = V(u_1, \ldots , u_s; v_1, \ldots , v_s)$$ obtained by taking the first *s* columns of *V*. By Proposition 7.3, $V^{\prime }$ is invertible, so $MV^{\prime }$ is also invertible. But $MV^{\prime }$ has a row of all zeroes (from taking the subvector of *x*). This shows that there must be at least r-s+1 non-zero entries in *x*. □

### Lemma 7.5

The Jukes-Cantor model satisfies the rank property *RP*(*r*, *k*) for $r \le 2^{k-1} - k + 1$.

### Proof

Let *r* and *k* satisfy $r \le 2^k - k+1$. We must show that for any trees $T_1, \ldots , T_r$ on *k* leaves, and generic probability distributions $P_i \in M^\mu _{T_i}$, the matrix

$$ \begin{pmatrix} \textrm{Flat}_{[n-1]|n}(P_1)&\textrm{Flat}_{[n-1]|n}(P_2)&\cdots&\textrm{Flat}_{[n-1]|n}(P_r) \end{pmatrix} $$

has rank rκ. However, by the fact that the set of matrices with rank $\le \alpha$ is Zariski closed, it suffices to show that there is a single choice of parameters that gives the rank 4*r*.

Suppose that *T* is a tree. Note that if we set the transition matrix on an edge to be the identity matrix (which is a transition matrix in the Jukes-Cantor model), that will give a probability distribution on a tree obtained from *T* by contracting the corresponding edge. Hence, if we set the transition matrices of all internal edges of the tree to be the identity matrix, this will give us distributions on the star tree. So distributions on the star tree appear as distributions in the model on any tree. Thus the result follows if we prove the Lemma when $T_1 = T_2 = \cdots = T_r$ are all the star trees.

To this end, let $T_1, \ldots , T_r$ be star trees with *k* leaves, and consider the matrix

$$ M = \begin{pmatrix} \textrm{Flat}_{[k-1]|k}(P_1)&\textrm{Flat}_{[k-1]|k}(P_2)&\cdots&\textrm{Flat}_{[k-1]|k}(P_r) \end{pmatrix}. $$

Our first step is to apply the Fourier transform to the parametrization. Let $$Q_i$$ be the Fourier transformation of the probability distribution $$P_i$$. The Fourier transform is linear, and it transforms the matrix *M* into a new matrix $M^{\prime }$ which is

$$ M' = \begin{pmatrix} \textrm{Flat}_{[k-1]|k}(Q_1)&\textrm{Flat}_{[k-1]|k}(Q_2)&\cdots&\textrm{Flat}_{[k-1]|k}(Q_r) \end{pmatrix}. $$

The new matrix $M^{\prime }$ is obtained from *M* by row and column operations, so *M* and $M^{\prime }$ have the same rank.

Now we analyze the parameterization in the Fourier coordinates. For the Jukes-Cantor model on a star tree $T_i$ we have that

$$ q(g_1, \ldots , g_k) = {\left\{ \begin{array}{ll} \prod _{i = j}^k a^{(i, j)}_{g_j} & \sum _{j = 1}^k g_j = 0 \\ 0 & \text{ otherwise } \end{array}\right. } $$

where we have a set of parameter $a^{(i,j)}_g$ for each tree $T_i$, each edge *j*, and each group element g∈G. For the Jukes-Cantor model we have that

$$ a^{(i,j)}_{(1,0)} = a^{(i,j)}_{(0,1)} = a^{(i,j)}_{(1,1)} $$

for all *i* and *j*. We can rearrange rows and columns of $M^{\prime }$ so that is has a block form, grouping all the columns by the value of $$g_k$$, and grouping the rows so that, for a fixed value of $$g_k$$ we have all the $$(g_1, \ldots , g_{k-1})$$ so that $$q(g_1, \ldots , g_k) \ne 0$$ together.

After this rearrangement of rows and columns, $M^{\prime }$ will be a block diagonal matrix, with 4 blocks, each block of size $$4^{k-2} \times r$$. We need to show that each of these blocks has full rank, so we get that the total rank is 4*r* as desired.

To show that the block matrices have the appropriate rank, we note that each such matrix is a generalized Vandermonde matrix. Indeed, each entry of the matrix is a monomial, and each column is an identical copy of the first column, but with new variables. Note, however, that the condition $a^{(i,j)}_{(1,0)} = a^{(i,j)}_{(0,1)} = a^{(i,j)}_{(1,1)}$ will yield repeated monomials in each column. Thus to complete the proof, we need to figure out how many distinct monomials there are, so we can apply Proposition 7.3.

Consider the map from

$$ \phi : \mathbb {Z}_2 \times \mathbb {Z}_2 \rightarrow \{0,1\}, \phi (g,h) = {\left\{ \begin{array}{ll} 0 & \text{ if } g = h = 0 \\ 1 & \text{ otherwise }. \end{array}\right. } $$

Then two monomials $q(g_1, \ldots , g_k)$ and $q(h_1, \ldots , h_k)$ are identical if and only if $\phi (g_i) = \phi (h_i)$ for all *i*. So we just need to count the number of equivalence classes for each fixed value of $g_k$.

This is straightforward to do: if $\phi (g_k) = 0$, then $(\phi (g_1), \ldots , \phi (g_{k-1}))$ can be any string in $\{0,1\}^{k-1}$ except the strings that have exactly one 1. There are $2^{k-1} - k + 1$ such strings. On the other hand, if $\phi (g_k) \ne 0$ then $(\phi (g_1), \ldots , \phi (g_{k-1}))$ can be any string in $\{0,1\}^{k-1}$ except the string with all zeroes. There are $2^{k-1} - 1$ such strings. We need to take the smaller of these two values to get a consistent rank across all the blocks. Hence this shows that the Jukes-Cantor model satisfies *RP*(*r*, *k*) with $r \le 2^{k-1} - k + 1$. □

### Remark 7.6

The proof of Lemma 7.5 can be extended to the case of the fully symmetric model, in the case where $\kappa \ge 3$, by using the Fourier transform over the group $\mathbb {Z}_\kappa$, instead of $\mathbb {Z}_2 \times \mathbb {Z}_2$.

### Corollary 7.7

Any 4-state equivariant model satisfies the rank property *RP*(*r*, *k*) with $r \le 2^{k-1} - k + 1$. In particular, the Kimura 2-parameter model, the Kimura 3-parameter model, and the Strand Symmetric model all satisfy the rank property *RP*(*r*, *k*) with $r \le 2^{k-1} - k + 1$.

### Proof

We just need to show the existence of a single choice of parameters that give the desired rank condition. However, since the Jukes-Cantor model is a submodel of all of those other models, the result of Lemma 7.5 gives the desired result. □

Note that the bound $$r \le 2^{k-1} - k + 1$$ is not best possible for those other models besides the Jukes-Cantor model. Each model would require a more careful analysis to improve the results. Following the proof, for the group-based models, it suffices to determine the number of distinct monomials of different types in the block structure of the matrix $M^{\prime }$. Both Kimura 2-parameter and 3-parameter models will have significantly more distinct monomials than the Jukes-Cantor model, and so the rank property will hold for larger values of *r*.

Now we proceed to prove the Extended rank property for the Jukes-Cantor model. Again, that will also give a result for other models, though it is probably not the best possible for K2P, K3P, SSM.

### Lemma 7.8

The Jukes-Cantor models satisfies the extended rank property *ERP*(*r*, *k*) for $r \le 2^{k-1} - k+ 1$.

### Proof

Let $T_1$ be a tree on k+1 leaves that does not contain the split $[k-1]| \{k, k+1\}$, and any trees $T_2, \ldots , T_r$ on *k* leaves, and generic probability distributions $P_i \in M^\mu _{T_i}$. Consider the matrix the matrix

$$ M = \begin{pmatrix} \textrm{Flat}_{[k-1]|\{k, k+1\}}(P_1)&\textrm{Flat}_{[k-1]|k}(P_2)&\cdots&\textrm{Flat}_{[k-1]|k}(P_r) \end{pmatrix}. $$

We must show that *M* has rank $4(r-1) + 4^2$, generically.

As in the proof of Lemma 7.5, it suffices to prove that there is a single choice of parameters that achieves the desired rank. Then we can set many parameters equal to identity matrices, and consider the resulting trees that arise by contracting those edges. To that end, we can assume that trees $T_2, \ldots , T_r$ are all *k* leaf star trees.

As in the proof of Lemma 7.5, we apply the Fourier transform to all probability distributions to get a matrix

$$ M' = \begin{pmatrix} \textrm{Flat}_{[k-1]|\{k, k+1\}}(Q_1)&\textrm{Flat}_{[k-1]|k}(Q_2)&\cdots&\textrm{Flat}_{[k-1]|k}(Q_r) \end{pmatrix}. $$

Looking at the final blocks we have the matrix

$$ \begin{pmatrix} \textrm{Flat}_{[k-1]|k}(Q_2)&\cdots&\textrm{Flat}_{[k-1]|k}(Q_r) \end{pmatrix}. $$

This is the same matrix we have seen in the proof of Lemma 7.5, with one fewer set of columns. So it has rank 4(r-1) since JC has the rank property RP(r-1,k) with these values of *r* and *k*. Furthermore, after reordering rows and columns, as in the proof of Lemma 7.5, it can be broken into 4 blocks, each of which is a generalized Vandermonde matrix.

The 4 generalized Vandermonde matrices from the previous paragraph each have size $$(r-1) \times 4^{k-2}$$, and those generalized Vandermonde matrices each have full rank. We want to use Lemma 7.4 to complete the proof. In particular, we will show that there is a choice of parameters for the tree $$T_1$$ so that the resulting matrix $$\textrm{Flat}_{[k-1]|\{k, k+1\}}(Q_1)$$ has the property that each of its four vectors that it contributes to the support of one of the four Vandermonde submatrices from the previous paragraph has at most 4 nonzero entries. This will ensure that $M^{\prime }$ has the correct rank, by applying Lemma 7.4. With these observations in mind, we consider various restriction on the tree $$T_1$$ and matrices $$\textrm{Flat}_{[k-1]|\{k, k+1\}}(Q_1) $$ that can be produced.

First of all, $T_1$ must have a split that is not compatible with $[k-1] | \{k, k+1\}$. After relabeling the leaves, we can assume this split has the form

$$ A_j | B_j = \{1, \ldots , j-1, k\}| \{j, \ldots , k-1, k+1\} $$

for some *j* between 2 and k-1. We can assume that $T_1$ is the tree with only this one split $A_j|B_j$ as an internal split, by setting all other internal edges to have an identity matrix as the transition matrix.

For the edges in the tree $T_1$, we will make special choices for the Fourier parameters. For all the pendant edges except those going to leaves 1, *k*, and k+1, we set the Fourier parameter $a^i_{(0,0)} = 1$ and all other Fourier parameters $a^i_g$ equal to zero. Note that this does not correspond to biologically relevant choice of parameters, but this does not matter for our algebraic proof. For the pendant edges associated to the leaves 1, *k*,  and k+1, we set $a^i_g = 1$ for all $g \in \mathbb {Z}_2 \times \mathbb {Z}_2$. Finally, for the edge corresponding to the split $A_j|B_j$, we choose generic values for the parameters.

Consider a vector $(g_1, \ldots , g_{k+1}) \in (\mathbb {Z}_2 \times \mathbb {Z}_2)^{k+1}$. We break $(g_1, \ldots , g_{k+1})$ into blocks $(g_1, \textbf{h}_1, \textbf{h}_2, g_{k}, g_{k+1} )$, where

$$ \textbf{h}_1 = (g_2, \ldots , g_{j-1}) \quad \textbf{h}_{2} = (g_{j}, \ldots , g_{k-1}). $$

The Fourier coordinate $q(g_1, \textbf{h}_1, \textbf{h}_2, g_{k}, g_{k+1})$ will be nonzero if and only if

1. $$g_2 = g_3 = \cdots = g_{k-1} = (0,0)$$
2. $$g_1 + g_{k} + g_{k+1} = (0,0)$$.

In the event that this is satisfied, the corresponding Fourier coordinate is given by the formula

$$ q(g_1, \textbf{h}_1, \textbf{h}_2, g_{k}, g_{k+1}) = a^{A_j|B_j}_{g_{k+1}}. $$

With this structure in mind, we can investigate the location of nonzero entries in the matrix $\textrm{Flat}_{[k-1]|\{k, k+1\}}(Q_1)$. As in the Proof of Lemma 7.5, we reorder the rows. We do this so there are four blocks of rows, the blocks determined by the value

$$ g_1 + g_2 + \cdots + g_{k-1}. $$

For the block indexed by the group element *g*, there are exactly 4 nonzero entries, corresponding to where

$$ g_1 = g, g_2 = \cdots = g_{k-1} = (0,0) $$

and $g_{k+1} = g + g_{k}$. Each of those nonzero entries is in a different row and column of the matrix $\textrm{Flat}_{[k-1]|\{k, k+1\}}(Q_1)$, and there are 4 in each of the blocks. Thus, we satisfy the conditions of Proposition 7.4, so *M* has rank $(r-1)4 + 4^2$. □

### Remark 7.9

The proof of Lemma 7.8 can be extended to the case of the fully symmetric model, in the case where $\kappa \ge 3$, by using the Fourier transform over the group $\mathbb {Z}_\kappa$, instead of $\mathbb {Z}_2 \times \mathbb {Z}_2$.

As in the case of the rank property, we also can see a similar result for the extended rank property, for the other equivariant models we have studied. We omit the proof which is the same as for the rank property. Again, these results are probably not best possible for those other models, and paying attention to the structure of the generalized Vandermonde matrices that arise in the other models can yield stronger results.

### Corollary 7.10

The Kimura 2-parameter model, the Kimura 3-parameter model, and the Strand Symmetric model all satisfy the extended rank property *ERP*(*r*, *k*) with $r \le 2^{k-1} - k + 1$.

## The No Shuffling Property

In this section, we prove that the models JC, K2P, K3P, and SSM satisfy the No Shuffling Property. Like the results from the previous section on the rank property and extended rank property, we are able to just show the result for the JC model and immediately deduce it for all other models.

Recall the No Shuffling Property, Definition 6.7. Note the No Shuffling Property does not have any conditions on *r*. The results should hold for every *r* (and we can of course, stop at r=κ). Also, note that if we prove the No Shuffling Property for a Markov model μ just for trees with 3 leaves, this will prove that the property also holds for trees with *n* leaves with n≥3 as well. Otherwise, there is some *n*, and trees $T_1, \ldots , T_r$ and generic distributions $P_i \in M^\mu _{T_i}$, where we form the matrix

$$ Q = \begin{pmatrix} \pi _1 \textrm{Flat}_{[n-1]|n}(P_1)&\pi _2 \textrm{Flat}_{[n-1]|n}(P_2)&\cdots&\pi _r\textrm{Flat}_{[n-1]|n}(P_r) \end{pmatrix} $$

where $$\pi \in \Delta _r$$ be generic. Then we can form a new matrix $Q^{\prime }$ by taking κ columns of *Q*, and let *P* be the resulting tensor. If $$P \in M^\mu _T$$ for some *T*, then we can marginalize to three leaves and the same statement will be true for three leaves as well, contradicting that we had the No Shuffling Property for n=3. Hence, for the rest of this section, we will restrict to three leaf trees.

To show the No Shuffling Property, we will use phylogenetic invariants that come from the general Markov model, specifically certain commutation invariants described in Allman and Rhodes (2003); Strassen (1983).

### Theorem 8.1

Let *P* be a $\kappa \times \kappa \times \kappa$ tensor giving a distribution from the general Markov model with κ states on a 3 leaf tree with κ>2. For $i = 1, \ldots , \kappa$ let $P_{(i)}$ be the $\kappa \times \kappa$ matrix slice $P_{(i)} = ( P(i,u,v))_{u,v}$. Then

$$ P_{(i)} (\textrm{adj}(P_{(j)}) ) P_{(k)} - P_{(k)} (\textrm{adj}(P_{(j)}) ) P_{(i)} $$

for any *i*, *j*, *k*.

Note that $\textrm{adj}(A)$ denotes the classical adjoint of *A*, which is $\det (A) A^{-1}$ for an invertible matrix, and is well-defined even for singular matrices (since the entries are all polynomials in the entries of *A*).

### Lemma 8.2

Let μ be any phylogenetic Markov model on κ=4 states that contains the Jukes-Cantor model. Then μ satisfies the No Shuffling Property.

### Proof

We proved this result via a computation, and using the phylogenetic invariants from the general Markov model described in Theorem 8.1. Specifically, we generated three random tensors from the Jukes-Cantor model parameterization $P^1, P^2, P^3$. Then from those $4\times 4 \times 4$ tensors, we choose three matrix slices, and evaluate them in the polynomials from Theorem 8.1. We find that that this evaluates to zero only if the three matrix slices come from the same $P^i$.

The fact that the invariants from the General Markov Model evaluate to zero only when all three slices come from the same $P^i$ proves that all models that contain the Jukes-Cantor model will satisfy the No Shuffling Property. Indeed, if the No Shuffling Property is not satisfied for some model μ, then for all generic distributions from that model, there will be some choice of slices from different $P^1, P^2, P^3$ that satisfy the invariants that come from that model. The fact that the invariants vanish for the generic distributions in the model, implies that they vanish for all distributions that come from the model. Since the General Markov Model contains every phylogenetic model, the invariants for the General Markov model are contained in the ideal of invariants for any other model. Since we have produced a distribution in the Jukes-Cantor model that does not satisfy the phylogenetic invariants for the General Markov model with any non-standard set of slices, this proves that no model that contains the Jukes-Cantor model will satisfy those invariants for General Markov model. Hence any model containing the Jukes-Cantor model satisfies the No Shuffling Property. □

With the tools from Sects. 7 and 8 in hand, we are ready to apply Theorem 2.6 to deduce Corollary 2.7.

### Proof of Corollary 2.7

According to Theorem 2.6, to deduce Corollary 2.7, we must show that all of the equivariant models:

1. are standard Markov models,
2. satisfy the rank property $RP(2^k-k, k)$,
3. satisfy the extended rank property $ERP(2^k-k, k)$, and
4. satisfy the No Shuffling Property.

The fact that all the models are standard is the content of Lemma 7.1.

The JC model satisfies the rank property $RP(2^k-k, k)$ by Lemma 7.5. Any supermodel of the JC model will hence satisfy $RP(2^k-k, k)$ as well. The JC model satisfies the extended rank property $ERP(2^k-k, k)$ by Lemma 7.8. Any supermodel of the JC model will hence satisfy $ERP(2^k-k, k)$ as well. Finally, Lemma 8.2 shows that all the models that contain JC satisfy the No Shuffling Property. □
